# Fire mitigates bark beetle outbreaks in serotinous forests

**DOI:** 10.1007/s12080-021-00520-y

**Published:** 2021-07-08

**Authors:** Peter C. Jentsch, Chris T. Bauch, Madhur Anand

**Affiliations:** 1 Department of Applied Mathematics, University of Waterloo, 200 University Ave. W, Ontario Waterloo, Canada; 2 School of Environmental Sciences, University of Guelph, 50 Stone Rd E, Ontario Guelph, Canada

**Keywords:** Forest, Model, Dynamical, System, SIR, Wildfire, Fire, Pest, Mechanistic

## Abstract

Bark beetle outbreaks and forest fires have imposed severe ecological damage and caused billions of dollars in lost resources in recent decades. The impact of such combined disturbances is projected to become more severe, especially as climate change takes its toll on forest ecosystems in the coming years. Here, we investigate the impact of multiple disturbances in a demographically heterogeneous tree population, using an age-structured difference equation model of bark beetle outbreaks and forest fires. We identify two dynamical regimes for beetle and fire dynamics. The model predicts that fire helps dampen beetle outbreaks not only by removing host trees but also by altering the demographic structure of forest stands. We show that a stand thinning protocol, which reduces the population size of the largest few juvenile classes by a small percentage, is able to significantly reduce beetle-induced tree mortality. Our research demonstrates one approach to capturing compound disturbances in a mathematical model.

**Electronic supplementary material:**

The online version of this article (10.1007/s12080-021-00520-y) contains supplementary material, which is available to authorized users.

## Introduction

Ecosystems have long been characterized by resilience in the face of large disturbances such as fire, storms, pathogens, and drought, which are often interacting. For example, the pine forests of western North America are highly adapted to both wildfires and bark beetle infestations. Many pine species, including lodgepole (*Pinus contorta*) and ponderosa (*Pinus ponderosa*) pine, depend on stand replacing fires to maintain healthy populations in their endemic range Bentz et al. (2010).

Of the major natural processes influencing lodgepole pine forests, the two with the greatest potential for large scale disturbance are mountain pine beetle (MPB, *Dendroctonus ponderosae*) and fire Kaufmann et al. (2008). It has been noted that “In western North America, insect outbreaks and wildfires are the two most ecologically and economically significant natural forest disturbances” Meigs et al. (2016). The MPB is a small insect endemic to the pine forests of western North America. MPB has recently attained previously unrecorded outbreak levels, probably due to anthropogenic factors Bentz et al. (2010); Safranyik et al. (2007). British Columbia’s Ministry of Forests estimates that British Columbia contains roughly 35 million acres of lodgepole pine forest (about 23%) and slightly less ponderosa pine forest. They estimate that over the past 20 years, MPB has affected approximately 1.6 million acres of forest annually in British Columbia, more than forest fire and logging combined BC Ministry of Forest (2010).

Ecological studies examining the relationship between MPB and wildfire damage are numerous, but have not reached a strong consensus in all aspects Axelson et al. (2009); Lynch et al. (2006); Simard et al. (2011); Bradley and Tueller (2001); Kaufmann et al. (2008); Meigs et al. (2016); Agne et al. (2016); Seidl et al. (2016); Jenkins et al. (2008). Lynch et al. (2006) used remote sensing data associated with the 1988 Yellowstone National Park fires to investigate the link between fire prevalence and beetle attack. They found that beetle attack initially lowered the probability of crown fire in a patch, but bark beetle activity significantly increases fire risk 13-16 years in the future. On the other hand, Seidl et al. (2016) find that wildfire increases spatial variability in stands and therefore reduces the susceptibility of the stand to beetle outbreak. To make things more complicated, some studies have found that measures of burn severity are positively correlated with beetle damage Simard et al. (2011); Bradley and Tueller (2001), although the results of Simard et al. (2011) have been disputed by others Moran and Cochrane (2012).

MPB, and forest pests more generally, has attracted the attention of mathematical biologists since the 1970s due to importance of the problem and the dynamical complexity of outbreaks. The dynamical model of a full forest ecosystem would be intractable, necessitating simplifying assumptions. An early model of forest-pest dynamics by Ludwig et al. (1978) is a three-dimensional differential equation model derived from simple population dynamics principles by separating fast (pest dynamics) and slow (forest dynamics). Powell et al. (1996) derive a seven-dimensional nonlinear partial differential equation model, incorporating beetle pheromone dynamics, which they then integrate to a local ordinary differential equation model. Others look at just one facet of the forest ecosystem. For instance, since beetle lifecycle depends heavily on temperature, Gilbert et al. (2004) discussed three models which incorporate temperature-dependent emergence and attack. Tree mortality also exhibits sharp transitions as a function of tree vigour. Duncan et al. (2015) incorporated a Leslie matrix to explicitly model multiple vigour categories in a discrete time dynamical model, while Lewis et al. (2010) developed an infinite-dimensional model which accounts for arbitrary vigour distributions. Some recent research also considers dynamic interactions between forest pest outbreaks and human population decision-making regarding transport of infested campfire wood Barlow et al. (2014); Ali et al. (2015).

Whether fire suppression changes stand structure in a way that alters susceptibility to beetle attack is a current topic of research. It has been hypothesized that wildfire encourages variability in spatial structure Seidl et al. (2016), which inhibits the ability of the bark beetle to find hosts and therefore dampens outbreak dynamics. We hypothesize that demographic variability (in the age structure of tree populations) can have a similar effect on MPB outbreaks. Age structure is pertinent because MPB mortality is much higher among larger, and therefore older, trees Axelson et al. (2010); Safranyik et al. (2003). This aspect has been studied in at least two previous models of MPB Lewis et al. (2010); Duncan et al. (2015) and has been found to affect system dynamics, although the additional role of fire was not considered in these models. Our objective is to characterize the model dynamics of an age-structured tree stand subject to disturbance from both fire and bark beetles, and to understand how changes in stand age structure due to wildfire or control measures can influence bark beetle outbreaks.

## Methods

### Model description

Our model is based on a discrete-time model developed by Duncan et al. (2015), describing beetle-tree dynamics in a well-mixed, sufficiently large, single-species stand. We expand their model to include fire dynamics by introducing a category for burned trees, implemented as a Kermack–McKendrick-style contagious process Edelstein-Keshet (1988). We also add stochastic forcing to both the infested category and the burned category. The discrete-time dynamics are defined in terms of population size in the spring of year *n*. Trees killed by beetle infestation die over the course of a few years, becoming a snag (a dead or dying tree that remains standing), until they decompose enough that they no longer shade the forest floor. If a tree is infested in the summer of year *n*, its needles will turn red and it will be a “red snag” in the spring of year $n+1$. Then, in the spring of year $n+2$, a “grey snag” with grey needles. After this it will decay sufficiently that new juvenile trees can grow up in its place, in year $n+3$. Wildfire also produces snags: a tree that is standing and shading the forest floor but no longer alive. We assume that wildfire clears the forest faster than MPB infections, so a tree that has been sufficiently affected by fire in the summer of year of *n* becomes a snag in the spring of year $n+1$, and then the following spring, new juvenile trees come up in its place. We assume that the forest is at carrying capacity, so new trees can only come up at the locations where trees have died. The forest is assumed to be a monospecies lodgepole pine stand, which are common hosts of MPB in western Canada and the USA.

**Fig. 1:**
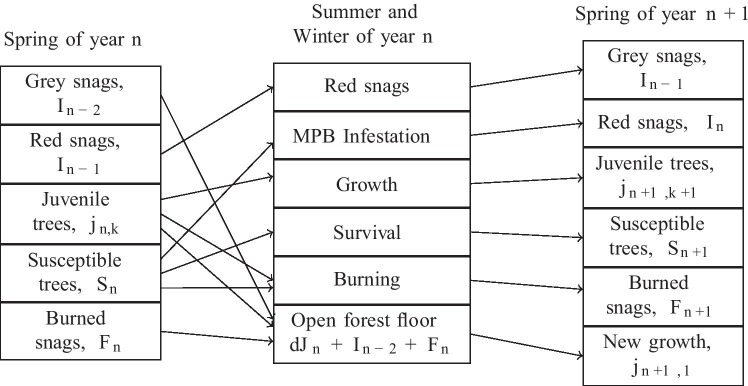
Conceptual diagram of compartments (state variables) and transitions between compartments across seasons. See main text and Table 1 for definitions of variables and parameters

Age structure is incorporated because beetles cannot effectively infest trees less than a certain diameter in size Safranyik et al. (2003); Alfaro et al. (2003). Juveniles grow through the *K* age categories, **at a rate of one age class per year**, with a probability $(1-d)$ of surviving until the next year. Figure 1 illustrates the cycle each category should move through in any particular year. We define the following state variables: $j_{n,k}$ is the population of juvenile trees of age *k* at year *n*, $J_n = \sum _{k = 0}^{K} j_{n,k}$, the total number of trees in the Juvenile class, $S_n$ is the population of susceptible trees at year *n*, $I_n$ is the population of infested trees at year *n*, and $F_n$ is the population of burned trees at year *n*.

The severity of forest fire in year *n* in the stand as a function of the previously unburned area is

1 $$\begin{aligned} P_n = T -\sum _{i=1}^{n} F_i e^{-\kappa (n - i)} \end{aligned}$$

where the variable $\kappa$ determines the half-life of decaying fuel. In other words, we define the severity or size of a fire in the year *n* as inversely proportional to the amount of land burned in recent seasons.

Then, our model is defined by:

2 $$\begin{aligned} j_{n+1, 1}= & {} d J_n + I_{n-2} + F_{n} \end{aligned}$$

3 $$\begin{aligned} j_{n+1, k}= & {} (1-d) j_{n, k - 1} - \frac{\alpha _1}{T} P_n j_{n,k-1 }, \nonumber \\ k= & {} 2 ... K-1 ,K \end{aligned}$$

4 $$\begin{aligned} S_{n+1}= & {} S_n + (1 - d) j_{n, K} - \left( I_n + \frac{\alpha _2}{T} P_n I_n\right) \nonumber \\&- \frac{\alpha _2}{T}P_n \left( S_n + (1-d) j_{n, K}\right) - \sigma _F \gamma _n \end{aligned}$$

5 $$\begin{aligned} I_{n+1}= & {} r_1 I_n e^{-\beta _1 (T - S_{n+1})} - \frac{\alpha _2}{T} P_n I_n + \sigma _I \xi _n \end{aligned}$$

6 $$\begin{aligned} F_{n+1}= & {} P_n \left[ \frac{\alpha _1}{T} \sum _{k = 1}^{K-1} j_{n,k} + \frac{\alpha _2}{T}\left( S_n + (1 - d)j_{n,K}\right) + \frac{\alpha _2}{T}I_n\right] \nonumber \\&+ \sigma _F\gamma _n \end{aligned}$$

7 $$\begin{aligned} P_{n}= & {} T - \sum _{i=1}^{n} F_i e^{-\kappa (n - i)} \end{aligned}$$

$\xi _n$ and $\gamma _n$ are normal random variates with zero mean and unit variance, drawn independently in year *n*. The model conserves the total number of tree-equivalents, which is a tree, a snag, or an open space in the canopy where a tree will grow the next season. The conservation equation is $T = I_{n-1} + I_{n-2} + F_{n} + J_{n} + S_{n}$, which can also be seen as the left or right column in Fig. 1.A detailed explanation of these equations, and a proof that these equations conserves tree equivalents, appears in the Supplementary Information. Descriptions of the variables can be found in Table 1. Fire could have been modelled in a more complex way using a different timescale than seasonal beetle outbreaks, but we chose to simplify the modelling by matching the timestep of the pest outbreak cycles instead. Fire prevalence is also dependent on precipitation patterns, temperature, human activity, and other factors which operate on different time and spatial scales than our model. We assume this risk is roughly constant each year, and that it contributes to the environmental noise experienced by the system denoted by $\sigma _F$.

### Forest thinning protocol (FTP) and controlled burning protocol (CBP)

One of the MPB control methods is to thin the forest, or conduct controlled burns, to increase the overall resilience of the forest to outbreaks or wildfire Safranyik et al. (2001); Sartwell and Stevens (1975); Amman and Logan (1998). In this section we modify our discrete process to include a control protocol, which is a simplified description of altering the structure of a growing stand to limit susceptibility to MPB. Define $\tau$ as the fraction of juvenile trees removed from the *m* oldest juvenile age classes, each year. The removed trees are added to the youngest juvenile class, to model trees replaced by seedlings. Since it is not realistic to perform this every year, we also investigate the effect of performing this protocol every 5 years. We will refer to the preceding protocol as the *forest thinning protocol (FTP)*.

8 $$\begin{aligned} X_n = \arg \max _{S \subset [1,50]: |S| = m} \sum _{k \in S} j_{n,k} \end{aligned}$$

Let $X_n$ be the set of *m* largest juvenile age classes defined as in Equation 8. Mathematically, for all age classes $k' \in X_n$, we change Equation 3 to Equation 10. In order to thin the fraction $\tau$ of trees from each age class in $X_n$, we add the corresponding population to $j_{n,0}$.

9 $$\begin{aligned} j_{n+1,1}= & {} d J_n + I_{n-2} + F_n + \sigma _F \gamma _n \nonumber \\&+ \tau \sum _{k' \in X_n}\left( (1-d) j_{n, k' - 1} - \frac{\alpha _1}{T} P_n j_{n,k'}\right) \end{aligned}$$

10 $$\begin{aligned} j_{n+1, k'}= & {} (1-\tau )\left( (1-d) j_{n, k' - 1} - \frac{\alpha _1}{T} P_n j_{n,k' - 1}\right) , \nonumber \\&k'\in X_n \end{aligned}$$

11 $$\begin{aligned} F_{n+1}= & {} P_n \left[ \frac{\alpha _1}{T} \sum _{i = 1}^{K-1} j_{n,k} + \frac{\alpha _2}{T}\left( S_n + (1 - d)j_{n,K}\right) + \frac{\alpha _2}{T}I_n\right] + \sigma _F\gamma _n \nonumber \\&+ \tau \sum _{k' \in X_n}\left( (1-d) j_{n, k' - 1} - \frac{\alpha _1}{T} P_n j_{n,k' - 1}\right) \end{aligned}$$

Controlled burning is modelled similarly, but instead we add the reduced age compartments to the *F* compartment as shown in Equation 11. We will refer to this modification as the controlled burning protocol (CBP) in the text from here on.We only consider removing juvenile trees because a tree removed before it is susceptible to MPB has the most potential effect on reducing MPB infestation size.

### Parameters and simulation design

Table 1 contains a list of the parameters used in the model, their interpretation, and their baseline values. Duncan et al. used a similar model with parameters fitted to data as in Agne et al. (2016). We performed sensitivity analysis on all other parameters (including all fire-related parameters) as shown in the "Results" section.

**Table 1:** Parameters and baseline values of compound fire and pest model. Except for $\alpha _i$ and the noise magnitude, all parameters were obtained from Duncan et al. (2015)

Parameter name	Default value	Interpretation	Source
$r_1$	1.8	yearly fecundity of beetles	Powell and Bentz (2009)
$\beta _1$	$10.8 \times 10^{-6}$	search failure rate of MPB	Powell and Bentz (2009)
*d*	0.01	annual mortality rate of juveniles	Duncan et al. (2015)
$\alpha _1$	-	burning rate of juveniles	-
$\alpha _2$	-	burning rate of adult trees	-
$\kappa$	0.1	decay rate of fuel	-
*T*	110,000	total number of trees in stand	Powell and Bentz (2009)
*K*	50	number of juvenile generations	Duncan et al. (2015)
$\sigma _F$	20	noise in burned tree	
$\sigma _I$	20	noise in infested tree	
*m*	0	number of age classes considered by FTP and CBP	
$\tau$	0	fraction of juvenile trees removed from the *m* age classes with FTP and CBP	

To generate parameter planes, we simulated Equations 2-7 across a grid of parameter values. We conducted 100 simulations for each point on the parameter grid and computed the average outcome for that grid point. We also recorded a representative sample of the resulting time series. We found the dominant period of the outbreaks by finding the frequency with maximum modulus via the discrete Fourier transform of the time series. In the deterministic case (with no noise), this frequency is the period of the periodic solution. When noise is added and the system becomes stochastic, there is no longer a clear periodic solution, but it is possible to estimate the mean of the distribution of the period by averaging the dominant frequency of the system at equilibrium. The period is assumed to be 1, corresponding to a (stochastic) steady state, if the smallest and largest values of the susceptible time series were sufficiently close together. The model and analysis of model output were coded in Julia. Throughout the "Results" section, we mostly focus on the $\alpha _1,\alpha _2$ plane. We kept the remainder of the parameters constant as it was possible to set their values from empirical literature as described above.

## Results

We first characterize the dynamical regimes of the model as a function of the burning rates $\alpha _1$, $\alpha _2$, and the decay rate $\kappa$. Then, we describe how the forest responds to the FTP and CBP described previously. Note that the susceptible class refers to mature trees, i.e. those large enough to be susceptible to infestation by MPB. Maximum outbreak sizes and fire season sizes are taken over a 500 year period.

### Dynamical regimes

There are roughly two equilibrium dynamical regimes in the $\alpha _1, \alpha _2$ parameter plane, although the size of the equilibrium populations varies continuously with the parameters inside each dynamical regime. The shapes of these dynamical regimes are affected by the rate of fuel decay, $\kappa$.

**Fig. 2:**
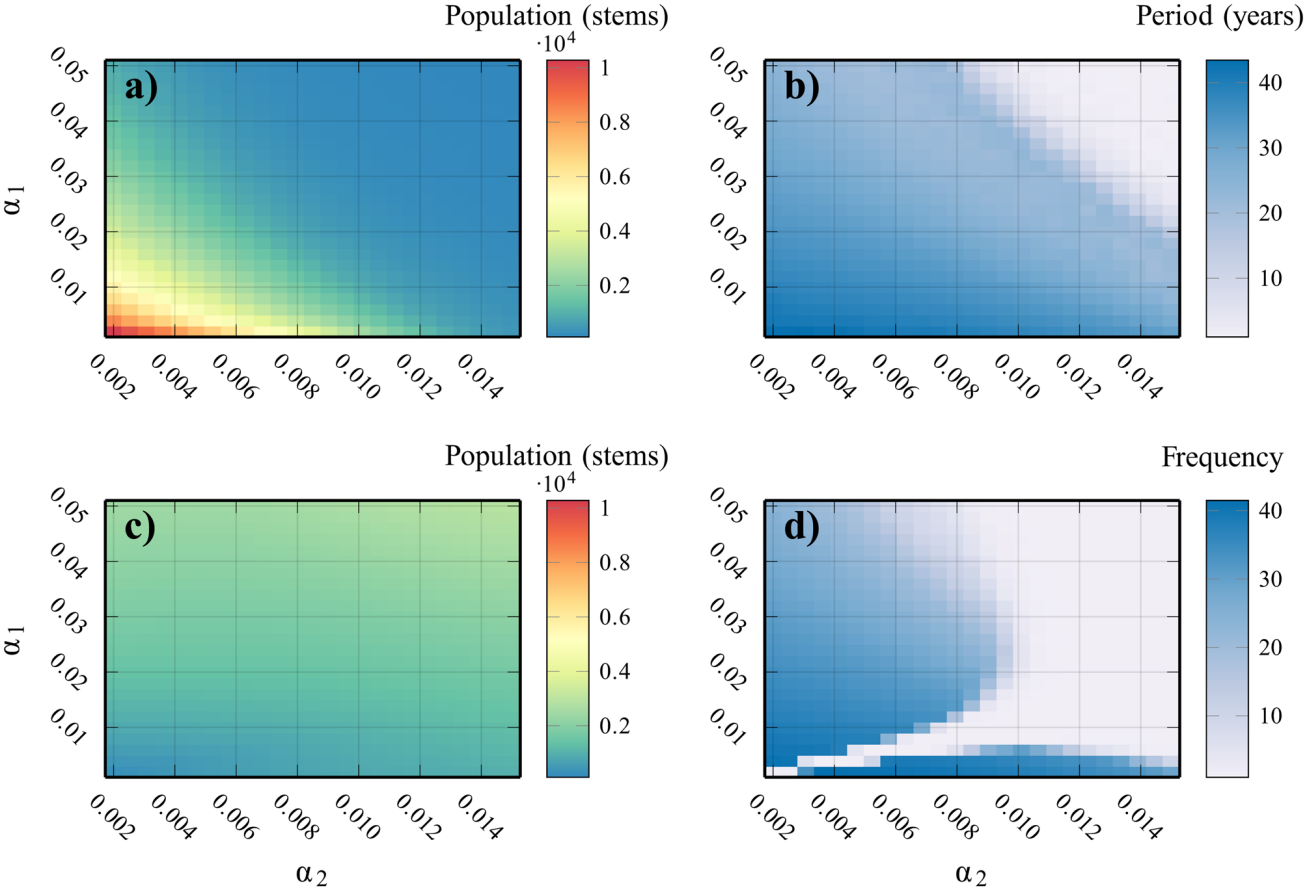
Approximate dynamical regimes of the system, where $\alpha _1$ is the burning rate of juvenile trees, and $\alpha _2$ is the burning rate of susceptible (mature) trees. **a**) Average size of largest MPB population (over 500 years at equilibrium) **b**) Average frequency of MPB outbreaks at equilibrium **c**) Average size of largest fire season (over 500 years at equilibrium) **d**) Average frequency of severe fire seasons at equilibrium. The juvenile burning rate ($\alpha _1$) and susceptible burning rate ($\alpha _2$) control fire and MPB prevalence. Large $\alpha _1,\alpha _2$ implies low infestation and also more regularity in the fire regime. All other parameters were set to baseline values (Table 1)

**Fig. 3:**
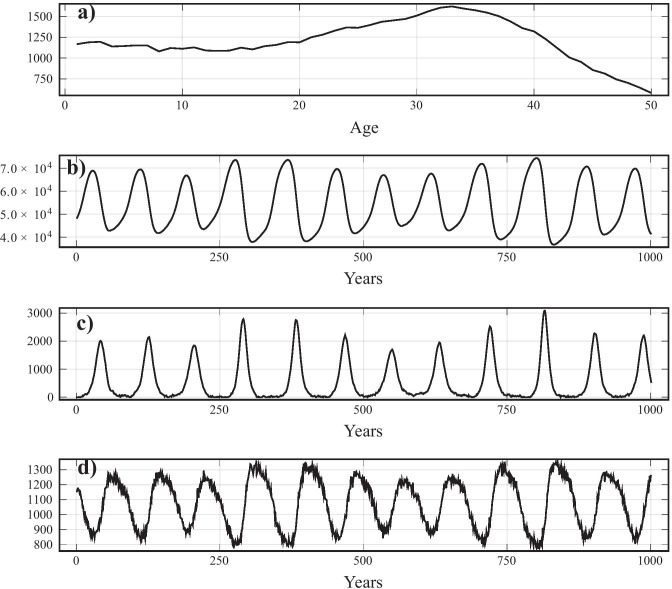
Time series of each state variable of a single realization where $\alpha _1 = 0.02$, $\alpha _2 = 0.0025$ , showing the regular outbreaks of pests, periodic shifts in fire prevalence, and uneven age distribution generated by MPB outbreaks. **a**) juvenile distribution at time $t=2050$ (note different x-axis), **b**) susceptible population after year *t*, **c**) infested tree population after year *t*, **d**) number of burned trees after year *t*. All other parameters were set to baseline values (Table 1)

As $\alpha _1, \alpha _2$ increase, the model displays larger, and more frequent fires, and smaller MPB outbreaks (Fig. 2). When $\alpha _1, \alpha _2$ are small and not equal, years with severe fire seasons roughly follow the same period as MPB outbreaks (Fig. 2b, d). The variation in fire season size is more pronounced when $\alpha _1$ is either much larger or smaller than $\alpha _2$. This is shown in by the unit period in Fig. 2d which signifies that the burned tree population is roughly constant and therefore recurs with period one. The presence of large stands with similar ages is determined by the size of the MPB outbreaks, since they mostly affect sufficiently old (susceptible) trees (Fig. 3a, b). This can been seen in the bump at $k = 40$, which will age out of the juvenile age classes and become a large concentration of susceptible trees, triggering an MPB outbreak. Dead trees from this MPB outbreak clear canopy space for seedlings, which causes another large even-aged stand to arise, and the cycle repeats.

### Impact of forest thinning protocol (FTP) and controlled burning protocol (CBP)

The model predicts that the FTP described in "Forest thinning protocol (FTP) and controlled burning protocol (CBP)" (remove a fraction $\tau$ of trees in the *m* oldest juvenile age classes each year) is an effective way to control MPB outbreaks, as long as control intensity parameters are sufficiently large. We consider trimming fractions $\tau$ up to 0.15, and the number of age classes trimmed *m* up to 8.

The FTP reduces the size of MPB outbreaks differently depending on the values of $\alpha _1,\alpha _2$ (Fig. 6). The parts of the parameter regime where thinning is most effective at reducing MPB outbreak sizes occur when $\alpha _1$ is small, where we see approximately 70% smaller MPB outbreaks (Fig. 6a) for all considered values of $\alpha _2$. Generally, parameter ranges where MPB is more prevalent experience the largest reductions. With $\alpha _1 = 0.02$, $\alpha _2 = 0.0025$, there is a reduction in maximum outbreak population of about 30% when thinning the largest 8 stands by 15% each year (SI Fig. 1a). With $\alpha _1 = 0.01$, $\alpha _2 = 0.006$, MPB populations are already dampened by the fire regime, but MPB outbreak peak population sizes are reduced from roughly 1600 infested trees to 800 infested (SI Fig. 1b). A similar practice conducted every five years is almost as effective as the yearly trimming (Fig. 6b). Maximum MPB infestation sizes for FTP every 5 years and CBP are also in the SI Appendix. Increasing the heterogeneity of the age distribution in this way always reduces MPB populations by some amount. If we apply the CBP instead (see Equation 11), then controlled burns are largely effective with significant MPB populations, but can worsen outbreaks by up to 80% in regions were the MPB outbreak size is already small (Fig. 6c).

**Fig. 4:**
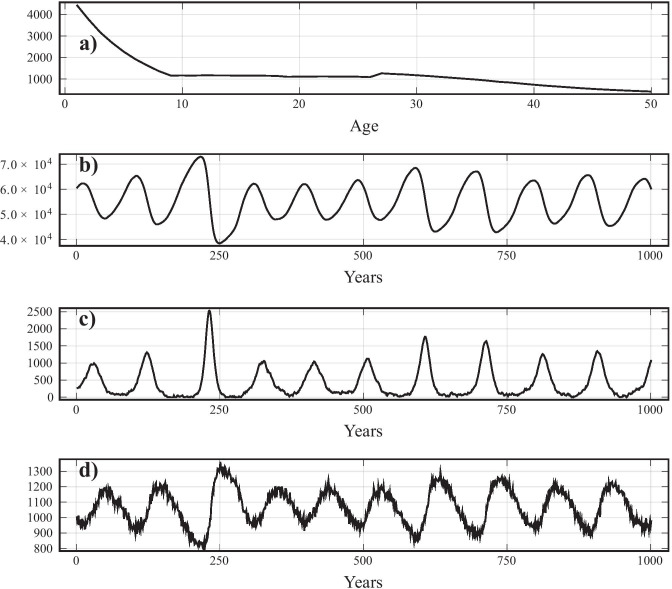
Time series showing realization of model under FTP with $\tau = 0.15$ fraction of $m = 8$ juvenile stands cleared, conducted each year, where $\alpha _1 = 0.02$, $\alpha _2 = 0.0025$. **a**) juvenile distribution at time $t=2050$ (note different x-axis), **b**) susceptible population after year *t*, **c**) infested tree population after year *t*, **d**) burned forest after year *t*. Notice the flattening of the age distribution compared to the same parameters with no FTP (Fig. 3)

**Fig. 5:**
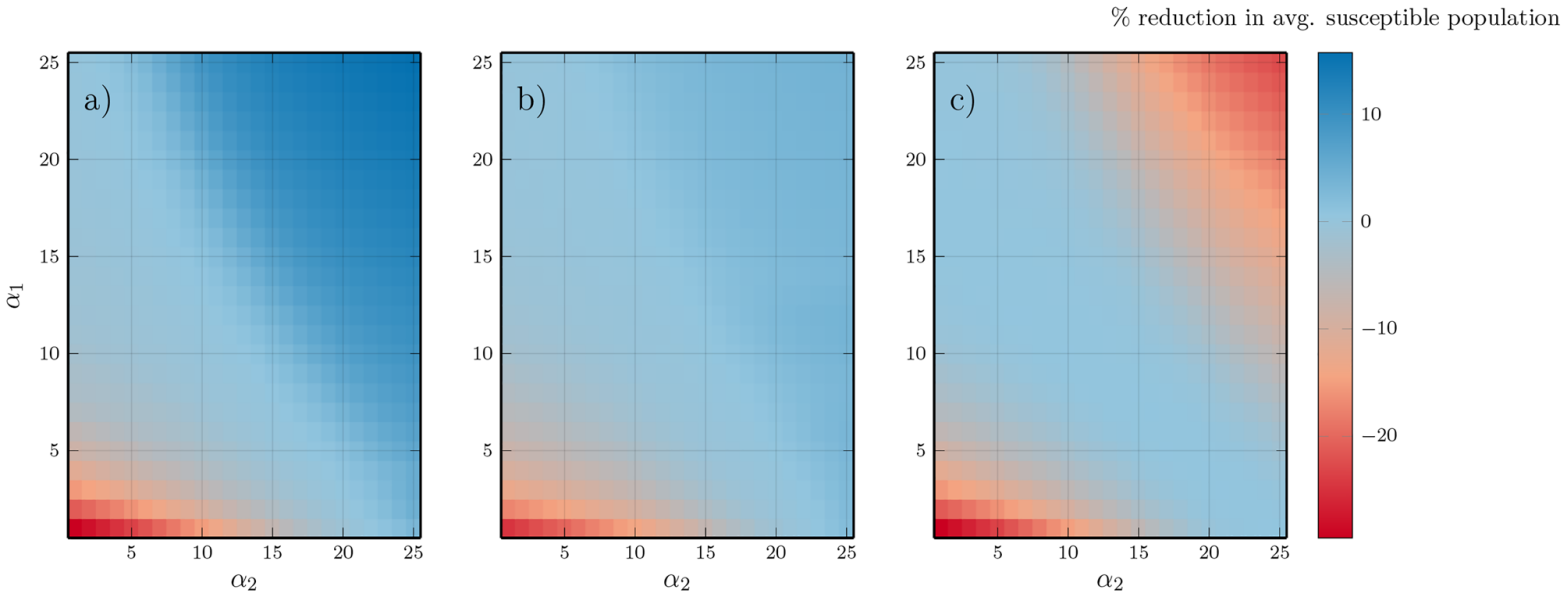
Percentage change in average susceptible (mature) forest population compared to no FTP with **a**) $\tau = 0.15, m = 8$, **b**) with $\tau = 0.15, m = 8$ applied every 5 years, **c**) controlled burning with $\tau = 0.15, m = 8$, with respect to burning rates $\alpha _1,\alpha _2$. FTP and CBP increase the average population of mature trees in many cases, in addition to reducing outbreak sizes

FTP, and to a lesser extent CBP, does not simply indirectly reduce the number of susceptible trees (and therefore available MPB hosts) but rather flattens the age distribution better to reduce the occurrence of large, even aged, stands. We compare the average susceptible population (Fig. 5) with and without FTP/CBP and find that in large parts of the parameter regime, the susceptible population is unchanged or increased, despite MPB outbreak sizes being reduced in most areas. Figure 4 shows a time series at the same parameters as Fig. 3, except with FTP flattening, to show the flattening of the age distribution.

**Fig. 6:**
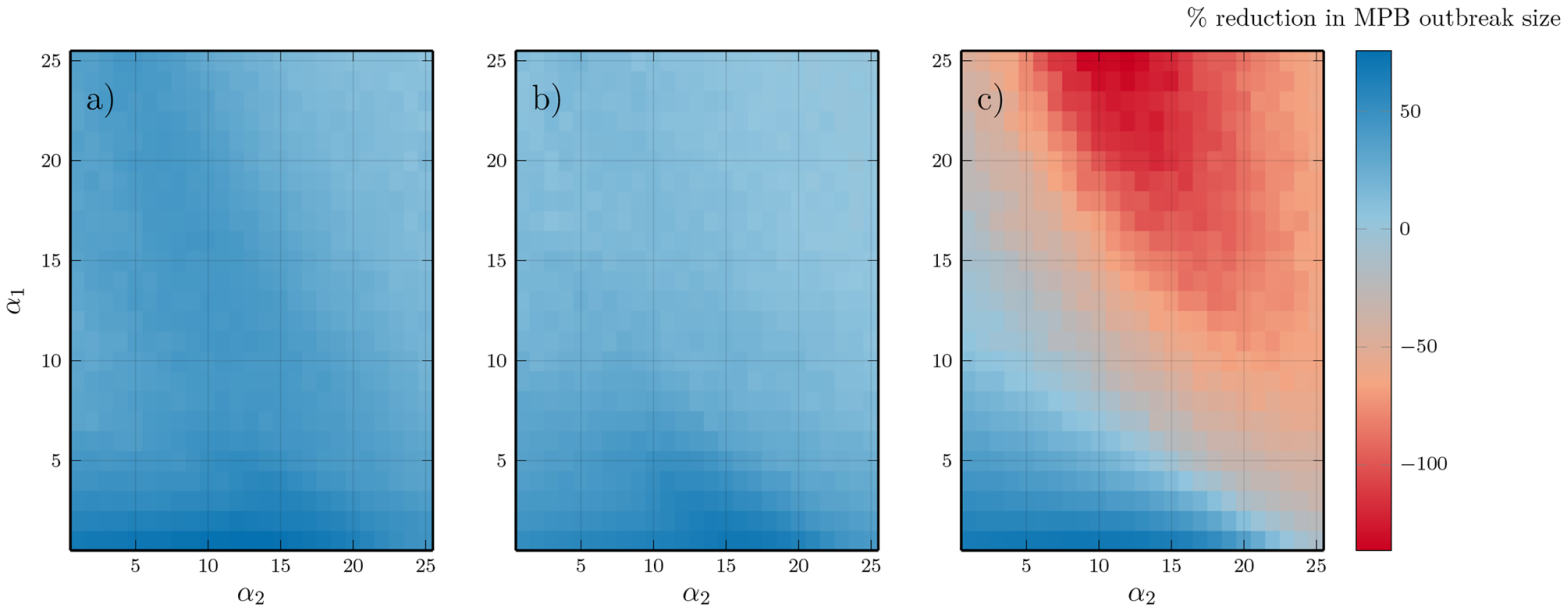
Percentage change in maximum MPB infestation size within 500 year period under FTP with **a**) $\tau = 0.15, m = 8$, **b**) with $\tau = 0.15, m = 8$ applied every 5 years, **c**) controlled burning with $\tau = 0.15, m = 8$, with respect to burning rates $\alpha _1,\alpha _2$. FTP is always effective at reducing MPB outbreaks, and CBP worsens them if the population of MPB is already low

## Discussion

In this paper we used a mathematical model of pest and fire dynamics in pine forests to show how fire can suppress beetle outbreaks. The effect is related not only to the assumption of competition between fire and beetles in the model, but also due to the impact of fire on the age structure of stands: fires remove many large, mature trees and make space for rapidly growing juvenile populations that are not susceptible to forest pest outbreaks. The behaviour of the fire–beetle system is due to the fact that susceptibility to fire cuts across all age classes, compared to beetle outbreaks that affect mostly mature age classes. We show that large outbreaks of wildfires and beetle outbreaks inhabit the same dynamical regime, and that very small beetle populations are consistent with a regular fire regime. These results echo ecological evidence from Kaufmann et al. (2008) and Seidl et al. (2016) showing that a consistent fire regime can dampen outbreaks of bark beetle in a serotinous forest stand. Furthermore, we showed how a stand thinning protocol can significantly reduce tree mortality due to MPB outbreaks in forests prone to both fire and beetle outbreaks. Only a small intensity of thinning is required to see significant results. Prescribed burning has a similar, although less significant, effect on the age structure of the forest and therefore similarly dampens MPB outbreaks. Prior to the arrival of European colonists, indigenous Americans routinely burned areas in western North America Barrett and Arno (1982), these practices were not recognized as beneficial by colonial governments, and were outlawed Boyd (1999).

### Implications for Fire/beetle management

Our work provides support for the practice of thinning forest stands to create more heterogeneity in age structure Jenkins et al. (2012); Negrón et al. (2017), despite the absence of spatially explicit dynamics in our model. We show that even small changes to the demographics of forest stands can result in large shifts in forest dynamics, dampening out oscillating disturbance patterns and thereby increasing stand resilience. Using an abstract model for this purpose hopefully allows the evidence to generalize better over the wide range of possible ecosystem parameters.

### Evidence in literature for dynamical regimes described

Broadly, our model can describe the current dynamical regime of stands of pine forests in the western interior with low fire susceptibility parameters $\alpha _1,\alpha _2$ (the bottom right-hand corner of Fig. 2a) depending on the location and time. Our model represents a single stand with autonomous parameters, and in reality, there are probably many possible dynamical regimes coexisting across the landscape and through time. Taylor et al. (2003) studied the wildfire and MPB history of interior British Columbia and also found this dynamic regime, albeit with decreasing prevalence of wildfire and increasing MPB outbreaks through the twentieth century. They find that the low frequency and severity of wildfire have increased the percentage of pine in susceptible age classes to 55%, consistent with our estimate for low $\alpha _1,\alpha _2$ (see Fig. 3a for an example of the large susceptible stands in this dynamical regime). Axelson et al. (2009) records that, for their study area in southern interior British Columbia, there has been a fire-free interval of over 100 years. While this period is much longer than in our model, a trend towards higher variance in fire periods does match our model for the aforementioned parameter range, and a more complex fire model could potentially capture this additional complexity. They also record an average return time of 36 years for MPB in their area, consistent with our estimate for sufficiently low burning parameters. Kulakowski et al. (2012) record a similar dynamic regime as Axelson et al. (2009) for the twentieth century, but more frequent fire outbreaks, more closely matching this model.

### Implications for Forest Ecosystems

The dry pine forest ecosystem that we model in this paper is home to many vertebrates who react to disturbance, biotic and otherwise, in different ways. Many wood-boring birds use MPB as a food source Norris and Martin (2008); Powell et al. (2002). Of these bird species, the three-toed woodpecker (*Picoides dorsalis*) and hairy woodpecker (*Leuconotopicus villosus*) depend significantly on bark beetles as a source of food Leatherman et al. (2012). Accounts estimate that they make up about 23% of the woodpeckers diet on an average year Beal (1911), although during outbreak years the fraction could be much larger Leatherman et al. (2012). Woodpeckers increase their reproduction rate during outbreak years of MPB Edworthy et al. (2011), so dampening MPB outbreaks could be detrimental to bird populations, although there are feedbacks here that warrant further study. At least one predator of MPB, the black-backed woodpecker (*Picoides arcticus*), is heavily dependent on wildfires for habitat. Therefore, improving forest heterogeneity would likely also improve resilience in woodpecker populations which depend on these disturbances for habitat. Small mammals that inhabit western pine forests differ on their preference for burn-cleared habitat Zwolak (2009). Mammals such as the deer mouse (*Peromyscus maniculatus*) strongly prefer burn clearing Zwolak and Foresman (2008), while the red backed-vole (*Myodes gapperi*) favours undisturbed stands Zwolak (2009). Increasing heterogeneity would improve the availability of both open stands for species which prefer the former habitat and closed, undisturbed stands for species which favour the latter. The impact of our results for these ecosystems is likely to be significant, but due to the complex feedbacks mentioned in these relationships, it is difficult to know without extending the model and further empirical data on the strength of these feedbacks.

The primary goal of this paper was to build on work on the age structured models of beetle-infested stands Duncan et al. (2015) to a dynamical situation with a more complex disturbance regime that includes wildfire, a common feature of the forests inhabited by MPB. The modelling of fire spread is a very complex problem which is dependent on many variables which are not modelled here. Moreover, the beetle infestation model we used was relatively simple, necessitating use of a simple fire model as well in order to retain tractability of the model. We opted for a simple approach derived from the compartmental modelling literature. The dynamics we see here are an average case, so a more sophisticated fire model would yield more detailed results. The assumed impact of fire on all age classes, and the mechanism through which we model fire spread could also be refined in future work. Snags are also not considered burnable material, which may have an effect on some of the dynamics. We chose not to include these to reduce the number of parameters, especially parameters for which we don’t have empirically-derived values. Lastly, the parameters which we drew from Duncan et al. Duncan et al. (2015) were not tested for sensitivity, and therefore, our findings could be affected by these values.

A number of other approaches that relax our simplifying assumptions could be explored in future research. Other models combine annual difference equations with continuous time intra-year equations Strohm et al. (2016); Lynch et al. (2006); Casagrandi and Rinaldi (1999). A continuous time summer phase is one way we could more accurately explicitly model a wildfire season. The FTP is straightforward and corroborates the findings of similar work with more complex mechanisms Strohm et al. (2016). Nevertheless, our control strategies could be significantly more detailed and take into account fire-regimes and current susceptible population. Our goal was to illustrate that we can take advantage of the system dynamics by flattening the age distribution through burning a small percentage of juvenile trees, but more complex strategies might be more efficient. Spatial models would provide even more possibilities for control options. We did not explore the complex relationship between bark beetle emergence and temperature. MPB life-cycles are heavily regulated by temperature: warm years can cause more than one generation to emerge in a season, and severe cold can wipe out large populations. The higher precipitation and temperatures predicted by models of climate change imply conditions more conducive to MPB reproduction and therefore MPB outbreaks. Fire season intensity is also affected by temperature, and some evidence suggests that increasing temperatures and earlier snowmelts are probably creating worse fire seasons in this area Westerling et al. (2006).

Serotinous forests will be subject to very different environmental regimes in coming decades that involve multiple stressors. We have demonstrated how a model can explore the impact of fire and control protocols on tree stand age structure and thus MPB outbreaks. Future models that account for multiple disturbance mechanisms could be useful for anticipating how forests will respond to novel environmental regimes in the rest of the twenty-first century.

## Electronic supplementary material

Below is the link to the electronic supplementary material.

Supplementary file1 (PDF 2763 kb)

## Data Availability

The source code for this work can be found at the author’s public git repository: https://git.uwaterloo.ca/pjentsch/fire-mitigates-bark-beetle-outbreaks-in-serotinous-forests
