# Supplementary material for: Fire mitigates bark beetle outbreaks in serotinous forests
Source: Theor Ecol. 2021 Jul 8;14(4):611–21. doi: 10.1007/s12080-021-00520-y (PMC8683088; doi:10.1007/s12080-021-00520-y)
Supplement: Supplementary file 1 — Supplementary file1 (PDF 2763 kb) [file 12080_2021_520_MOESM1_ESM.pdf]

1 Derivation of Model Equations

$$j_{n+1,1} = dJ_n + I_{n-2} + F_n \tag{1}$$

New juvenile trees are created each year according to equation 1. The number of juveniles of age 1 is equal to the total number of juveniles that died last year, $dJ_n = d\sum_{k=1}^{N} j_{n,k}$, plus the number of grey snags I_{-2}, plus the number of burnt snags (trees that burned the previous summer) F_n.

As mentioned in the main text, we use P_n to be the severity of forest fire in year n. It is defined by an exponentially decaying sum over the unburnt forest area from previous years, so P_n is not a state variable.

$$P_n = T - \sum_{i=1}^{n} F_i e^{-\kappa(n-i)} \tag{2}$$

The growth of juvenile trees is defined by equation 3. A fraction $1 - d$ of juveniles from class $k - 1$ grows into class k juveniles, minus the trees in this class that burn, proportional to P_n.

$$j_{n+1,k} = (1-d)j_{n,k-1} - \frac{\alpha_1}{T} P_n j_{n,k-1}, k = 2...K-1, K \tag{3}$$

The number of susceptibles in the spring of year $n + 1$ is equal to the number of susceptibles in the spring of year n, plus the number of juveniles growing into mature trees $((1 - d)jn, K)$. We subtract the trees that were infested in the summer of year n, I_n, the infested *and* burnt trees, $\frac{\alpha_3}{T} P_n I_n$, the trees that were only burnt, $\frac{\alpha_2}{T} P_n S_n$, and the juveniles from class K that would have become mature if they had not caught fire, $(\frac{\alpha_2}{T} P_n (1-d) j_{n,K})$.

$$S_{n+1} = S_n + (1-d)j_{n,K} - (I_n + \frac{\alpha_3}{T} P_n I_n) - \frac{\alpha_2}{T} P_n (S_n + (1-d)j_{n,K}) \tag{4}$$

$$I_{n+1} = r_1 I_n e^{-\beta_1 (T - S_n - (1-d)j_{n,K} + (I_n + \frac{\alpha_2}{T} P_n I_n) + \frac{\alpha_2}{T} P_n (S_n + (1-d)j_{n,K})} - \frac{\alpha_3}{T} P_n I_n \tag{5}$$

The model for infested trees is based on ricker-style dynamics, where r_1 is the reproduction rate of beetles, and the exponential term denotes the probability that each individual will find a susceptible tree. The number of infested trees burned is subtracted after reproduction, for simplicity. As in Duncan et al., we have $I_{n+1} = r_1 I_n e^{I_n + I_{n-1} + F_n + J_{n+1}}$, where the exponent is the number of current non-susceptible trees. From the conservation of tree-equivalents $T = I_n + I_{n-1} + F_n + J_{n+1} + S_{n+1}$, so the exponent is just $T - S_{n+1}$.

$$F_{n+1} = P_n \left[\frac{\alpha_1}{T} \sum_{k=1}^{K-1} j_{n,k} + \frac{\alpha_2}{T} (S_n + (1-d)j_{n,K}) + \frac{\alpha_3}{T} I_n \right] + \sigma_F \gamma_n \tag{6}$$

The burnt snags in the spring of year $n+1$ is equal to the sum of the number of burnt juveniles ($\frac{\alpha_1}{T} P_n \sum_i^{K-1} j_{n,K}$), susceptibles ($\frac{\alpha_2}{T} P_n(Sn + (1-d)j_{n,K})$), and infested ($\frac{\alpha_3}{T} I_n P_n$).

$$
\begin{aligned}
j_{n+1,1} &= dJ_n + I_{n-2} + F_{n-1} && \text{(7a)}\\
j_{n+1,k} &= (1-d)j_{n,k-1} - \frac{\alpha_1}{T} P_n j_{n,k-1}, \qquad k = 2...K-1, K && \text{(7b)}\\
S_{n+1} &= S_n + (1-d)j_{n,K} - \Big(I_n + \frac{\alpha_2}{T} P_n I_n\Big) - \frac{\alpha_2}{T} P_n \left(S_n + (1-d)j_{n,K}\right) - \sigma_F \gamma_n && \text{(7c)}\\
I_{n+1} &= r_1 I_n e^{-\beta_1 (T - S_{n+1})} - \frac{\alpha_2}{T} P_n I_n + \sigma_I \xi_n && \text{(7d)}\\
F_{n+1} &= P_n \left[\frac{\alpha_1}{T} \sum_{k=0}^{K-1} j_{n,k} + \frac{\alpha_2}{T} \left(S_n + (1-d)j_{n,K}\right) + \frac{\alpha_2}{T} I_n \right] + \sigma_F \gamma_n && \text{(7e)}\\
P_n &= T - \sum_{i=1}^{n} F_i e^{-\kappa(n-i)} && \text{(7f)}
\end{aligned}
$$

The following lemma demonstrates that the model equations preserve the total tree equivalent population present in the initial conditions.

Lemma 1 *Let $I_{-1} + I_{-2} + F_0 + J_0 + S_0 = T$. The equation $I_{n-1} + I_{n-2} + F_n + J_n + S_n = T$ is true for all $n \geq 0$ under the evolution equations 7a-7f.*

Proof First, notice that the only individuals leaving the Juvenile compartment J_n are the surviving oldest juvenile age class $(1-d)j_{n,K}$ and the sum of the trees burned from each juvenile age class (except the oldest), $\frac{\alpha_1}{T} P_n \sum_{k=1}^{K-1} j_{n,k}$. The individuals entering the Juvenile compartment are the seedlings germinating in the canopy gaps created by the gray snags I_{n-2} and the trees burned the previous summer, F_n. Therefore we have the following equation for the total number of juvenile trees J_{n+1}.

$$
J_{n+1} = J_n - (1-d)j_{n,K} + I_{n-2} + F_n - \frac{\alpha_1}{T} P_n \sum_{k=1}^{K-1} j_{n,k} \tag{8}
$$

Then, proceed by induction. The base case, $I_{-1} + I_{-2} + F_0 + J_0 + S_0 = T$, is true by definition.

For the inductive step, assume that $I_{n-1} + I_{n-2} + F_n + J_n + S_n = T$ is true, then we have:

$$
I_{n-1} + I_{n-2} + F_n + J_n + S_n + (1-d)j_{n,K} - (1-d)j_{n,K} = T \tag{9a}
$$

$$
\begin{aligned}
\implies J_n + I_{n-2} + F_n - (1-d)j_{n,K} + I_{n-1} + S_n + (1-d)j_{n,K}&\\
+F_{n+1} - \frac{\alpha_1}{T} P_n \sum_{k=1}^{K-1} j_{n,k} - \frac{\alpha_2}{T} \left(S_n + (1-d)j_{n,K}\right) P_n&\\
- \frac{\alpha_2}{T} I_n P_n - \sigma_F \gamma_n = T&
\end{aligned} \tag{9b}
$$

where we use the definition of F_{n+1}.

$$\Longrightarrow J_{n+1} + I_{n-1} + S_n + (1-d)j_{n,K} + F_{n+1} - \frac{\alpha_2}{T}\left(S_n + (1-d)j_{n,K}\right)P_n - \frac{\alpha_2}{T} I_n P_n - \sigma_F \gamma_n = T \tag{9c}$$

$$\tag{9d}$$

by definition of J_{n+1}.

$$\Longrightarrow J_{n+1} + I_{n-1} + S_n + (1-d)j_{n,K} + F_{n+1} - \frac{\alpha_2}{T}\left(S_n + (1-d)j_{n,K}\right)P_n - \frac{\alpha_2}{T} I_n P_n - \sigma_F \gamma_n + I_n - I_n = T \tag{9e}$$

$$\tag{9f}$$

$$\Longrightarrow J_{n+1} + S_{n+1} + F_{n+1} + I_n + I_{n-1} = T \tag{9g}$$

where we obtain the conservation equation for the spring of year $n+1$ by definition of S_{n+1}. Therefore the inductive step is true, and the model equations conserve tree equivalents. □

2 Initial conditions

The initial conditions we use in the model are defined as follows.

$$I_0 = 2000 \tag{10a}$$
$$I_{-1} = 1000 \tag{10b}$$
$$I_{-2} = 0 \tag{10c}$$
$$S_0 = 108000 \tag{10d}$$
$$F_0 = 1000 \tag{10e}$$
$$j_{0,k} = 0, \qquad k = 1...K \tag{10f}$$
$$\tag{10g}$$

These conditions were chosen to provide reasonable representations of the system being modeled. The model appears to be robust to the choice of initial conditions and so the observations in the main text hold for any reasonable set of initial conditions. Note that $I_{-1} + I_{-2} + F_0 + J_0 + S_0 = 110000 = T$, where T is the parameter that determines the total number of stems, from the main text.

3 Additional Figures

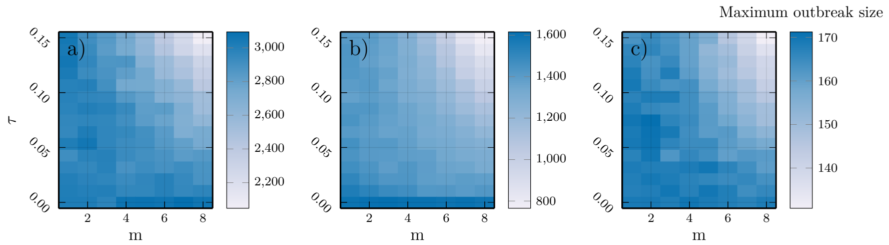

Fig. 1 Maximum MPB infestation size within 500 year period, under FTP with respect to τ fraction of m juvenile stands cleared. a) ($\alpha_1 = 0.02$, $\alpha_2 = 0.0025$), b) ($\alpha_1 = 0.01$, $\alpha_2 = 0.006$.), c)($\alpha_1 = 0.03$, $\alpha_2 = 0.0012$.)

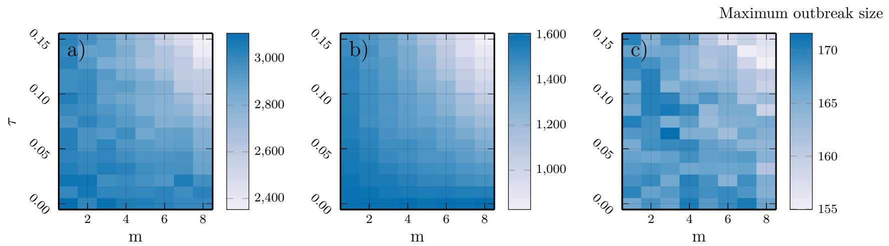

Fig. 2 Maximum MPB infestation size within 500 year period, under FTP with respect to τ fraction of m juvenile stands cleared, conducted every *five* years. a)($\alpha_1 = 0.02$, $\alpha_2 = 0.0025$), b)($\alpha_1 = 0.01$, $\alpha_2 = 0.006$.), c)($\alpha_1 = 0.03$, $\alpha_2 = 0.0012$.)

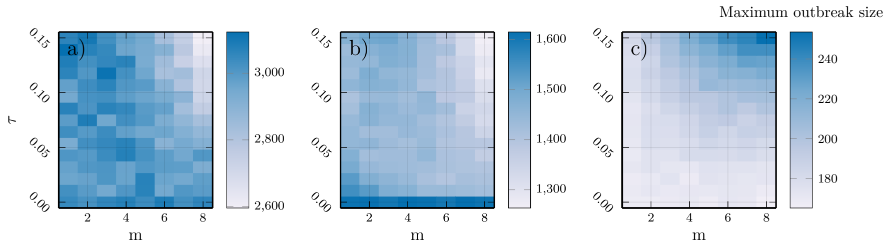

Fig. 3 Maximum MPB infestation size within 500 year period, under CBP with respect to τ fraction of m juvenile stands cleared, conducted each year. a)($\alpha_1 = 0.02$, $\alpha_2 = 0.0025$), b) ($\alpha_1 = 0.01$, $\alpha_2 = 0.006$.), c) ($\alpha_1 = 0.03$, $\alpha_2 = 0.0012$.)
